# Short-term Effects of Outdoor Air Pollution on Lung Function among Female Non-smokers in China

**DOI:** 10.1038/srep34947

**Published:** 2016-10-13

**Authors:** Yun Zhou, Yuewei Liu, Yuanchao Song, Jungang Xie, Xiuqing Cui, Bing Zhang, Tingming Shi, Jing Yuan, Weihong Chen

**Affiliations:** 1 Department of Occupational & Environmental Health, School of Public Health, Tongji Medical College, Huazhong University of Science and Technology, Wuhan, Hubei 430030, China; 2 Key Laboratory of Environment and Health, Ministry of Education & Ministry of Environmental Protection, and State Key Laboratory of Environmental Health (Incubating), School of Public Health, Tongji Medical College, Huazhong University of Science and Technology, Wuhan, Hubei 430030, China; 3 Hubei Provincial Key Laboratory for Applied Toxicology, Hubei Provincial Center for Disease Control and Prevention, Wuhan, Hubei 430079, China; 4 Department of Respiratory and Critical Care Medicine, Tongji Hospital, Tongji Medical College, Huazhong University of Science and Technology, Wuhan, Hubei 430030, China; 5 Department of Immunization Program, Zhejiang Provincial Center for Disease Control and Prevention, Hangzhou, Zhejiang 310051, China

## Abstract

Short-term exposures to outdoor air pollutants have been associated with lower lung function, but the results are inconsistence. The effects of different pollutant levels on lung function changes are still unclear. We quantified the effects of outdoor air pollution exposure (NO_2_, PM_10_, O_3_, and PM_2.5_) on lung function among 1,694 female non-smokers from the Wuhan-Zhuhai Cohort in China by using linear mixed model. We further investigated the associations in the two cities with different air quality levels separately to quantify the effects of different pollutant level exposure on lung function. We found the moving averages of NO_2_, PM_10_, and PM_2.5_ concentrations were significantly associated with reduced FVC. In city at high pollutant level, the moving average of NO_2_, PM_10_, O_3_, and PM_2.5_ exposures were significantly associated with both FVC and FEV_1_ reductions. In the low-level air pollution city, PM_10_ (Lag03-Lag05) and O_3_ concentrations (Lag01-Lag03) were significantly associated with reduced FVC, while PM_10_ (Lag03-Lag05), O_3_ (Lag0-Lag03), and PM_2.5_ (Lag04-Lag06) exposure were significantly associated with reduced FEV_1_. Our results suggest that outdoor air pollution is associated with short-term adverse effects on lung function among female non-smokers. The adverse effects may persist for longer durations within 7 days at higher air pollutant levels.

Air pollution has drawn much public health concern globally, especially in developing countries. In China, it was reported that annual mean concentrations of PM_10_ and PM_2.5_ in 2014 were 105 μg/m^3^ and 62 μg/m^3^ respectively, which were 5 to 6 times more than those recommended by the World Health Organization (WHO) air quality guidelines for PM_10_ (20 μg/m^3^) and PM_2.5_ (10 μg/m^3^)^1^,^2^. Outdoor air pollution including particulate matter (PM), ozone (O_3_) and nitrogen dioxide (NO_2_) has been reported to be associated with short-term adverse effects on lung function in healthy adults, children, or patients with chronic obstructive pulmonary disease (COPD) or asthma^3^,^4^,^5^,^6^,^7^,^8^. However, most of these studies were conducted in populations in developed countries, where air pollutant levels were lower than developing countries (e.g. China). A few studies have examined the short-term effects of relatively high outdoor air pollutant level on lung function alteration among school children or college students^9^,^10^,^11^,^12^,^13^,^14^,^15^,^16^, the effects of high air pollution level exposure is still unclear among adults. In addition, the association between air pollution and lung function is inconsistence. Some studies reported that the negative association could be weak or absent for longer moving average exposure^3^,^17^, while a Brazil study reported that a cumulative adverse effects on lung function^16^. Whether the effects of different pollutant levels on lung function are different is still unknown.

Another barrier to explore the association between air pollution and lung function is the existence of effect modifiers. One is cigarette smoking, which can lead to significant lung function decline through pulmonary oxidant stress, inflammation and tissue damage^18^. Another potential effect modifier is sex. Lung function levels among females are significantly lower than those among males due to lung development and physiology^19^. Both smoking and sex have been reported to modify the association between outdoor air pollutants and lung function, but the results remain inconsistent; a few studies surprisingly did not find the effect modification^3^,^20^,^21^. Nonetheless, it is necessary and important to identify the effect modification either by stratified analysis or by restricting study populations.

In this study, we investigated short-term effects (up to 7 days) of air pollutants (NO_2_, PM_10_, O_3_, and PM_2.5_) on lung function among restricted to lifetime female non-smokers from the Wuhan-Zhuhai Cohort. We further examined the associations in the two study cities with different air quality levels separately to quantify the effects of different pollutant level exposures on lung function.

## Results

**Figure 1:**
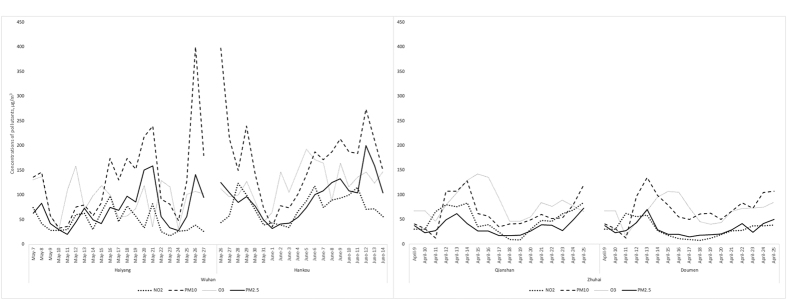
Distribution of ambient air pollutants in two cities during the preceding 0–7 days (Lag0-Lag7) before lung function test. The plotted values are the 24-h averages of pollutant concentrations reported from the 4 monitoring stations in two cities.

Characteristics for the study participants are presented in Table 1. The mean age of all subjects was 56.0 years. There were no significant differences in height, BMI, cooking and asthma between participants from Wuhan and Zhuhai (all *p* > 0.05). Percentage of passive smokers, FVC and FEV_1_ were significantly lower in Zhuhai city than those in Wuhan, while age, heart disease, physical activities, and FEV_1_/FVC were significantly higher in Zhuhai compared with those in Wuhan (all *p* < 0.05). Air pollutant distributions and their Spearman correlation coefficients during the days before lung function test are given in Fig. 1, and Tables 2 and 3. The concentrations of NO_2_, PM_10_, O_3_, and PM_2.5_ in Zhuhai were lower than those in Wuhan. Pairwise correlations for each pair of the 4 air pollutants were significantly positive (all *p* < 0.01). The positive correlations were found between NO_2_ and PM_10_, NO_2_ and PM_2.5_, and PM_2.5_ and PM_10_ in both Wuhan and Zhuhai (all *p* < 0.05). O_3_ was positively correlated with PM_10_ in Zhuhai (Spearman correlation coefficient r = 0.39, *p* = 0.02). Distributions and Spearman correlation coefficients for the different moving averages of pollutant exposures were shown in Supplementary Table S1. Positive correlations were observed between each pair of the moving averages of the 4 pollutants exposure (Lag0-Lag07) among all participants (All *p* < 0.01).

**Figure 2:**
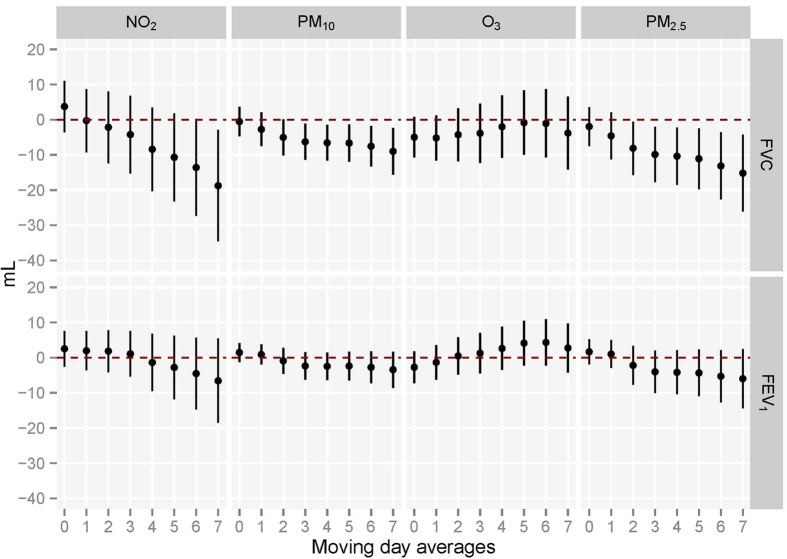
Associations between moving averages of air pollutant exposures before test and lung function (N = 1,694). Abbreviations: FVC, forced vital capacity; FEV_1_, forced expiratory volume in 1 second. The linear mixed models included city (Wuhan and Zhuhai) as a random effect and adjusted for age, height, body mass index, passive smoking status, asthma, heart diseases, physical activities and cooking meals at home. Associations with lung function are scaled per 10 μg/m^3^ increase in all the pollutants concentrations for each moving average.

Figure 2 shows the associations between per 10 μg/m^3^ increase in all the pollutants concentrations for each moving average and lung function alteration using single models among all the 1,694 subjects. NO_2_ (Lag07), PM_10_ (Lag03-Lag07) and PM_2.5_ (Lag02-Lag07) were significantly associated with decreased FVC, and no pollutant exposures were observed to be associated with FEV_1_. The significantly negative association between exposure to pollutants and lung function (FVC and FEV_1_) were stronger for longer moving averages of exposures. We observed that negative effects of NO_2_, PM_2.5_ and PM_10_ on lung function become stronger for longer moving averages of exposures. The influence of air pollutants on FVC was stronger than those on FEV_1_. Furthermore, we also quantify the associations between each interquartile ranges-increase air pollutant exposures and lung function (See Supplementary Fig. S1) (IQRs for NO_2_, PM_10_, O_3_ and PM_2.5_ were 43.2, 94.8, 52.7 and 57.4, respectively). We observed that each IQR-increase of NO_2_,. PM_10_ or PM_2.5_ was significantly associated with a 81.04, 85.27 or 87.18 ml decline of FVC in the 8-day moving average of exposures (Lag07).

We examined the effects of air pollutants (Lag07) on lung function for different groups (Table 4). Age was a potential modifier in association between O_3_ exposure and FVC decline (*p* values for interaction = 0.04). The adverse effect of O_3_ on lung function was stronger in participants aged more than 45 year-old than those aged under 45 years old.

**Figure 3:**
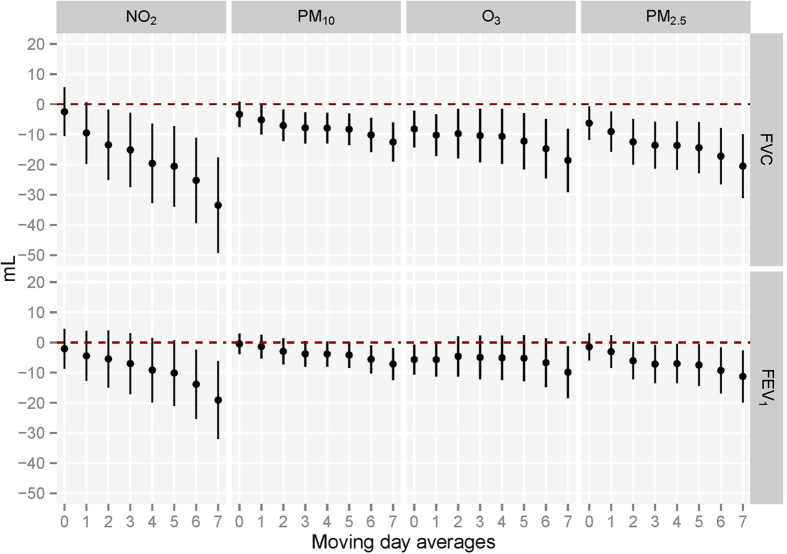
Associations between moving averages of air pollutants exposures before test and FVC and FEV_1_ with single pollutant models in Wuhan (N = 1,177). Abbreviations: FVC, forced vital capacity; FEV_1_, forced expiratory volume in 1 second; NO_2_, nitrogen dioxide; O_3_, ozone; PM_10_, particulate matter <10 μm in diameter; PM_2.5_, particulate matter <2.5 μm in diameter. *Estimated change is calculated by linear regression models with adjustment for age, height, body mass index, passive smoking status, asthma, heart diseases, physical activities and cooking meals at home. Associations with lung function are scaled per 10 μg/m^3^ increase in all the pollutants concentrations for each moving average.

**Figure 4:**
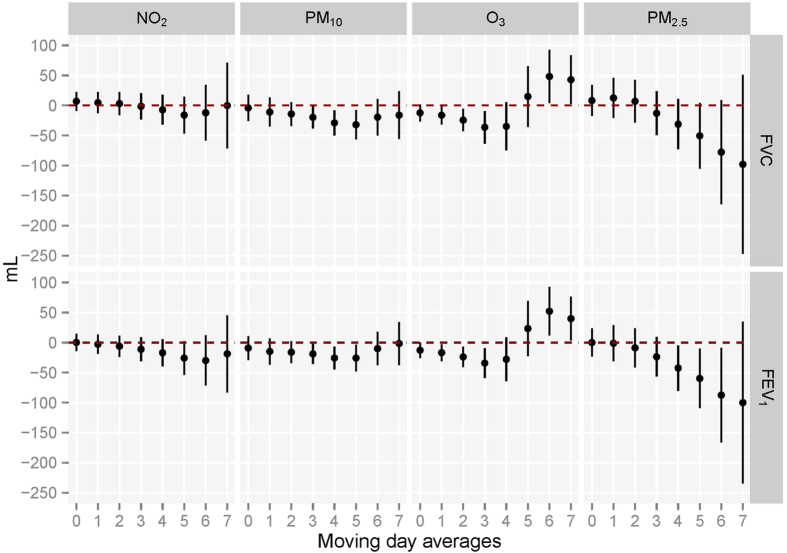
Associations between moving averages of air pollutants exposures before test and FVC and FEV_1_ with single pollutant models and Zhuhai (N = 517). Abbreviations: FVC, forced vital capacity; FEV_1_, forced expiratory volume in 1 second; NO_2_, nitrogen dioxide; O_3_, ozone; PM_10_, particulate matter <10 μm in diameter; PM_2.5_, particulate matter <2.5 μm in diameter. *Estimated change is calculated by linear regression models with adjustment for age, height, body mass index, passive smoking status, asthma, heart diseases, physical activities and cooking meals at home. Associations with lung function are scaled per 10 μg/m^3^ increase in all the pollutants concentrations for each moving average.

Figure 3 provides the associations between the moving averages of the 4 air pollutant exposures and lung function levels among non-smoking females living in Wuhan. Short-term exposures of NO_2_ (Lag02-Lag07), PM_10_ (Lag01-Lag07), O_3_ (Lag0-Lag07), and PM_2.5_ (Lag0-Lag07) were significantly associated with FVC reduction. NO_2_ (Lag06 and Lag07), PM_10_ (Lag06-Lag07), O_3_ (Lag0, Lag01 and Lag07), and PM_2.5_ (Lag03-Lag07) were significantly associated with reduced FEV_1_. The associations were stronger for longer moving averages of exposures. In Zhuhai, PM_10_ (Lag03-Lag05) and O_3_ exposure (Lag01-Lag03) was significantly associated with FVC reduction, while PM_10_ (Lag03-Lag05), O_3_ (Lag0-Lag03), and PM_2.5_ (Lag04-Lag06) exposures were significantly associated with FEV_1_ reduction, and the associations became weak or absent for longer moving averages of exposures (Fig. 4).

## Discussion

In this study, we found that NO_2_ (Lag07), PM_10_ (Lag03-Lag07), and PM_2.5_ (Lag02-Lag07) were significantly associated with FVC reduction among female non-smokers. We also found that air pollution levels may modify the short-term effects of air pollutant exposures on lung function. The adverse effects of high air pollutant levels on lung function could cumulate over several days, while the effects of low air pollutant levels could appear on the same day as exposure, and weak or absent for longer moving averages of exposures.

We noted that the associations of air pollutant exposures with lung function alterations were different between two cities at different pollutant levels. One possible reason for the different changes of lung function between Wuhan and Zhuhai might be that air pollutant concentrations in two cities are various. In China, an individual score (IAQI) is assigned to the level of each pollutants and the final air quality index (AQI) is the highest of those 6 scores. Air quality is divided into 6 categories according to the AQI level, including 0 to 50, 51 to 100, 101 to 150, 151 to 200, 201 to 300, and more than 300, which represents excellent, good, lightly, moderately, heavily or severely polluted level of air pollution, respectively. According to the report from China’s Ministry of Environmental Protection, in 2014, the number of days at different polluted levels (lightly, moderately, heavily or severely polluted) in Wuhan was 183 (50.1%), while only 44 (12.1%) in Zhuhai annually. Compared with Wuhan, the air pollutant concentrations in Zhuhai city are much lower and closer to those in some developed countries. Similarly, the associations in Zhuhai were consistent to the results of most studies in developed countries at low pollutant levels. A cohort study in the US reported acute adverse effects of PM_2.5_, NO_2_ and O_3_ (Lag01-Lag02) were associated with FEV_1_ and FVC decline^3^. A Swiss cross-sectional study also reported acute exposure (Lag0 and Lag03) to total suspended particulate were significantly associated with decreased FVC and FEV_1_ among non-smokers^17^. In contrast, only a few studies examined the associations between relatively high air pollutant and lung function. A Brazil study at high air pollutant levels (PM_10_: 84.68 μg/m^3^, NO_2_: 92.50 μg/m^3^ and O_3_: 81.08 μg/m^3^) reported that increased moving averages of PM_10_ (Lag01-Lag02) exposure were associated with lung function decline among school children. They also observed a cumulative adverse effects on lung function^16^. Nonetheless, the Brazil study did not detail the associations for more than 3 days moving averages of pollutant exposure. In this study, we confirmed and extended the results of the Brazil study by investigating up to 7 days moving averages of exposure. We found that high levels of PM_10_, NO_2_, O_3_ and PM_2.5_ might affect lung function on the same day as the exposure and cumulate almost a week. It was hypothesized that compensatory protective mechanisms were responsible for lung function recovery after low concentrations of air pollutant exposures^22^. Whereas several day cumulative exposures to high levels of air pollutants may cause lung function decrement beyond compensatory and even the impairment in lung, which is likely difficult to relieve or dismiss.

The component of air pollution is another possible reason for the different effects on lung function in two cities. During the study period, the primary pollutants in Wuhan and Zhuhai were PM and O_3_, respectively. As a secondary pollutant, ground-level O_3_ is generated when emissions like nitrogen oxide and volatile organic compounds produced by cars, factories and other sources baked in the hot summer sun. Zhuhai is at downwind position of Guangzhou and Dongguan city, where the air creates high levels of O_3_, especially in summer.

In this study, we also noted that current and previous day exposure of O_3_ was associated with both FVC and FEV_1_, indicating that adverse effects of O_3_ exposure on lung function occurs acutely. Animal evidences demonstrated that O_3_ exposure prime innate immunity and up-regulate expression of injury repair genes in the lung. Meanwhile, O_3_ can also stimulate airway neural receptors like airways C-fibers and transmit to the central nervous system through afferent vagal nerve pathways^23^,^24^, resulting in some airway narrowing, neural inhibition of inhalation effort at high lung volumes.

Similar to other studies, we found that outdoor PM was associated with lung function reduction. PM can induce small airways constriction by stimulating endothelia release and activating direct oxidant effects and inflammation^25^. Most studies suggest that PM can penetrates into and retains in the walls of small airways leading to generating free radicals and triggering intracellular oxidative stress. Free radicals can further recruit inflammatory cells and generate inflammatory mediators and then cause airway wall remodeling and lung tissue damage^26^,^27^,^28^,^29^,^30^. We also observed NO_2_ exposure was associated with lung function decline, similar to the results of previous studies^12^,^31^. Possible reasons for the observed reductions in spirometry parameters may be that the nitrate or nitrite formed from NO_2_ directly irritates or corrodes lung epithelial cells or tissue, increases the permeability of alveolar and capillary, leading to pulmonary edema. Inhalation of NO_2_ can also cause lung injury by inducing inflammation response and the imbalance of Th1/Th2 differentiation, and activating the JAK-STAT pathways^32^,^33^.

There are several strengths in this study. We chose two cities to investigate the short-term effects of outdoor air pollution on lung function at remarkably different air quality levels. In addition, we analyzed associations between air pollutant exposures and lung function among restricted samples, which can help avoid being confounded sex and cigarette smoking. Finally, we used the same methods for data collection in two different cities to avoid measurement biases, and all the results were adjusted for many potential confounders and predictors of lung function.

One limitation of our study is that we ignored indoor air pollution. The effects on lung function resulted from both outdoor and indoor air pollution exposures. Using outdoor air pollution as a proxy as the 24 hour exposure may cause exposure misclassification. However, previous studies suggested that outdoor air pollution measurements may be used as a surrogate for individual level exposures in most populations. Moreover, we did not collect data on temperature or humidity, and control them as potential confounders, though the two factors did not vary dramatically within each city during study period. It is also clear that both long-term and short-term effects of air pollution on lung function exist, but we are unable to study the long-term effect of air pollution and lung function because the air pollutant monitoring data were unavailable before 2013. Therefore we are unable to distinguish short-term and long-term effect of air pollution exposure on lung function in the study. Further studies are needed to explore the clearer separations of short-term and long-term effects of air pollution on lung function. In conclusion, female non-smokers may experience adverse effects on the respiratory system from air pollutants, and higher level may lead to continuous damage to the lung. To protect lung function in heavy polluted area, it is necessary for female non-smokers to lower indoor air pollution, take activities, and use personal protective equipment.

## Methods

### Study Population

The study population is from the Wuhan-Zhuhai cohort^34^, which was established between April 2011 and June 2012, and enrolled 4,812 residents aged 18 to 80 years who had lived in Wuhan (N = 3,053) or Zhuhai city (N = 1,759) for more than five years. Three years later, the cohort participants were followed up for personal information update and the second physical health examination. Due to lack of data on air pollutants during 2011 to 2012, we only included the lung function test data from the second physical health examination between 2014 and 2015. A face-to-face interview was conducted for each participant by trained investigators. Data on health and lifestyle questionnaires covered information on demographic characteristics, occupational hazards exposure, smoking history, passive smoking history, alcohol consumption, physical activity, and cooking were collected in the study. After interview, a physical examination including lung function test was conducted. Residents who refused to attend clinic visits, or had severe illnesses were excluded from the study. By excluding 877 males and 653 cigarette smokers, 1,694 female non-smokers were included in this analysis.

### Ethics Statement

The research protocol was approved by the institutional review boards of Tongji Medical College, Huazhong University of Science and Technology, P.R. China. The methods were carried out in accordance with the relevant guidelines. All participants enrolled in this study gave written informed consent for participation.

### Lung function test

Lung function test was performed by specialists using electronic spirometers (Chestgraph HI-101, CHEST Ltd., Tokyo, Japan). Each participant was done in a sitting position with a nose clip after at least 5 minutes of normal breathing, and advised not to smoke for at least one hour or not to eat a large meal for two hours before the test. False teeth should be left in place unless they prevented the participant from forming an effective seal around the mouth piece. Three acceptable volume-time curves of forced vital capacity (FVC) or forced expiratory volume in one second (FEV_1_) were obtained and recorded in accordance with the American Thoracic Society (ATS) recommendations^35^.

### Environmental air pollution exposure assessment

Daily air pollution data on NO_2_, PM_10_, O_3_, and PM_2.5_ concentrations in Wuhan and Zhuhai were obtained from the National Real-Time Air Quality Monitoring Data Publishing Platform developed by China National Environmental Monitoring Center (CNEMC). There were 10 and 4 available fixed-sit air quality monitoring stations in Wuhan and Zhuhai, respectively. All the monitoring stations were located away from traffic, industrial sources, buildings or residential sources of emissions from the burning coal waste or oil, according to technical guidelines of the Chinese government^36^. Air pollutant levels for the four study communities (2 in Wuhan, and 2 in Zhuhai) were assessed by using data from the nearest monitoring stations. For each study community, average daily values for each pollutant were calculated using its hourly ambient air concentration. The analytical methods and instruments for air pollutants were conducted according to the ambient air quality standards in China. The concentration of PM_2.5_ or PM_10_ was measured by using the micro oscillating balance method and/or the β absorption method. NO_2_ and O_3_ were measured by using the chemiluminescence method (and/or differential optical absorption spectroscopy) and ultraviolet fluorescence method (and/or differential optical absorption spectroscopy) respectively.

### Statistical analysis

Daily average air pollutant concentrations were matched with lung function data for each participant before analysis. Spearman correlation coefficient was used to evaluate the bivariate associations between air pollutants during the study period. Associations between exposure variables and lung function were assessed using linear mixed models by including city (Wuhan/Zhuhai) as a random effect. The cumulative effects were examined by modelling moving average concentrations during the preceding 0–7 days (Lag0-Lag07) before lung function test. For example, Lag01 represented a 2-day moving average exposure, which was calculated as the average concentrations of the current and the previous day. Potential confounders were adjusted in all linear models, including age, height, body mass index (BMI), heart disease, asthma, occupational hazard exposure, passive smoking status, drinking status, physical activity, and cooking. Associations were quantified by using estimated changes and 95% confidence intervals (CIs) of lung function (FVC and FEV_1_) levels by each 10 μg/m^3^ increase of air pollutant concentrations.

We also stratified the analyses in different groups including age groups (<45 and ≥45 years old), BMI groups (<24 and ≥24 kg/m^3^), regular physical activity groups (yes and no) and cooking groups (yes and no) by linear mixed models. Effect modification of each covariate in association of each pollutant and lung function was calculated by including an interaction term of each pollutant multiplied by the covariate in the linear mixed model.

We further tested whether association between each air pollutant exposure and lung function (FVC and FEV_1_) differed with two cities at different polluted levels by using linear regression. All statistical analyses were performed using the SAS 9.3 software. The statistical significant level was defined as *p* < 0.05 (2-sided).

## Additional Information

**How to cite this article**: Zhou, Y. *et al.* Short-term Effects of Outdoor Air Pollution on Lung Function among Female Non-smokers in China. *Sci. Rep.* **6**, 34947; doi: 10.1038/srep34947 (2016).

## Supplementary Material

Supplementary Information

## Figures and Tables

**Table 1:** Characteristics of all non-smoking females in Wuhan and Zhuhai (N = 1,694).

Characteristics	Total	Wuhan	Zhuhai	*p* value
N (%)	1,694	1,177	517	—
Age, year (Mean ± SD)	56.0 ± 12.0	55.4 ± 12.5	57.2 ± 10.6	0.003
Height, cm (Mean ± SD)	153.7 ± 5.7	153.8 ± 5.8	153.4 ± 5.7	0.55
Body mass index, kg/m^2^, (Mean ± SD)	24.8 ± 3.6	24.8 ± 3.7	24.6 ± 3.4	0.87
Passive smoking, N (%)				<0.001
No	957 (56.5)	617 (52.4)	340 (65.8)	—
Yes	737 (43.5)	560 (47.6)	177 (34.2)	—
Cooking, N (%)				0.10
No	246 (14.5)	182 (15.5)	64 (12.4)	—
Yes	1448 (85.5)	995 (84.5)	453 (87.6)	—
Asthma, N (%)				0.61
No	1689 (99.7)	1173 (99.7)	516 (99.8)	—
Yes	5 (0.3)	4 (0.3)	1 (0.2)	—
Heart disease*, N (%)				0.003
No	1658 (97.9)	1160 (98.6)	498 (96.3)	—
Yes	36 (2.1)	17 (1.4)	19 (3.7)	—
Physical activity, N (%)				<0.001
No	725 (42.8)	566 (48.1)	159 (30.8)	—
Yes	969 (57.2)	611 (51.9)	358 (69.2)	—
FVC, ml (Mean ± SD)	2369.9 ± 530.5	2436.2 ± 545.2	2219.0 ± 461.4	<0.001
FEV_1_, ml (Mean ± SD)	2006.1 ± 462.1	2026.1 ± 471.3	1960.7 ± 437.5	0.007
%FEV_1_/FVC	84.8 ± 8.6	83.3 ± 8.6	88.2 ± 7.5	<0.001

Abbreviations: FVC, forced vital capacity; FEV_1_, forced expiratory volume in 1 second; SD, standard deviation.

*The 10th version of the International Classification of Diseases (ICD-10) was used to classify heart diseases (ICD-10 codes: I00–I09, I11, I13, and I20–I51).

**Table 2:** Distribution of all pollutants (daily average of ambient air pollutants).

	Total (N = 75)				Wuhan (N = 41)				Zhuhai (N = 34)			
	Mean	SD	Range	Median	Mean	SD	Range	Median	Mean	SD	Range	Median
NO_2_, μg/m^3^	50.73	28.40	7.75 to 124	43.2	60.36	29.04	17.6 to 124	57.6	39.13	23.08	7.75 to 84.50	32.5
PM_10_, μg/m^3^	112.32	78.06	12.3 to 400.4	84.0	149.76	85.19	33 to 400.4	146.0	67.17	32.04	12.30 to 134.75	60.63
O_3_, μg/m^3^	93.20	38.67	16.0 to 193	89.0	108.05	40.31	30 to 193	110.8	75.30	27.91	16.0 to 142.50	72.8
PM_2.5_, μg/m^3^	60.71	41.25	15.0 to 200	45.4	83.57	42.19	20.3 to 200	77.4	33.14	15.03	15.0 to 72.50	27.1

Abbreviations: NO_2_, nitrogen dioxide; O_3_, ozone; PM_10_, particulate matter <1 μm in diameter; PM_2.5_, particulate matter <2.5 μm in diameter; SD, standard deviation.

**Table 3:** Spearman correlation coefficients among air pollutants.

Air pollutants	Total (N = 75)				Wuhan (N = 41)				Zhuhai (N = 34)			
	NO_2_	PM_10_	O_3_	PM_2.5_	NO_2_	PM_10_	O_3_	PM_2.5_	NO_2_	PM_10_	O_3_	PM_2.5_
NO_2_, μg/m^3^	1				1				1			
PM_10_, μg/m^3^	0.59*	1			0.53*	1			0.37^†^	1		
O_3_, μg/m^3^	0.37*	0.51*	1		0.31	0.28	1		0.23	0.39^†^	1	
PM_2.5_, μg/m^3^	0.69*	0.90*	0.44*	1	0.54*	0.94*	0.25	1	0.80*	0.64*	0.21	1

Abbreviations: NO_2_, nitrogen dioxide; O_3_, ozone; PM_10_, particulate matter <10 μm in diameter; PM_2.5_, particulate matter <2.5 μm in diameter.

*p < 0.01; ^†^*p *< 0.05.

**Table 4:** The estimated changes in FVC and FEV_1_ associated with 10 μg/m^3^ increase in all the pollutants at lag07 in different groups (N = 1,694).

Lung function parameters	Stratified characteristics	Estimated change in Lung function*			
		NO_2_	PM_10_	O_3_	PM_2.5_
FVC					
	Age				
	<45	−44.78 (−92.35, 2.80)	−8.58 (−25.29, 8.13)	−16.62 (−45.46, 12.21)	−15.61 (−43.20, 11.98)
	≥45	−26.40 (−42.17, −10.62)	−11.95 (−18.63, −5.27)	−12.49 (−23.01, −1.97)	−20.59 (−31.58, -9.60)
	*p* value for interaction	0.23	0.69	0.04	0.66
	Body Mass Index				
	<24	−22.42 (−47.10, 2.26)	−14.95 (−25.33, −4.57)	1.14 (−14.86, 17.14)	−24.48 (−41.30, −7.66)
	≥24	−26.06 (−45.01, −7.11)	−6.07 (−13.88, 1.74)	−16.86 (−29.34, −4.39)	−10.91 (−23.90, 2.09)
	*p* value for interaction	0.37	0.87	0.09	0.96
	Cooking				
	Yes	−35.53 (−51.19, −19.87)	−13.23 (−19.76, −6.71)	−16.85 (−27.16, −6.54)	−23.15 (−33.95, −12.35)
	No	4.77 (−25.10, 34.63)	1.65 (−11.99, 15.28)	0.31 (−22.67, 23.29)	3.85 (−16.37, 24.07)
	*p* value for interaction	0.94	0.76	0.82	0.80
	Exercise				
	Yes	−31.40 (−51.24, −11.56)	−12.65 (−21.14, −4.17)	−14.09 (−27.16, −1.02)	−21.02 (−34.94, −7.11)
	No	−15.23 (−38.82, 8.36)	−5.85 (−15.42, 3.71)	−7.75 (−23.23, 7.73)	−11.60 (−27.38, 4.18)
	*p* value for interaction	0.31	0.18	0.79	0.24
FEV_1_					
	Age				
	<45	−30.12 (−68.79, 8.56)	−8.55 (−22.02, 4.91)	−3.33 (−26.46, 19.79)	−17.94 (−40.38, 4.51)
	≥45	−12.11 (−24.47, 0.24)	−4.39 (−9.57, 0.80)	−3.11 (−10.69, 4.46)	−7.74 (−16.28, 0.80)
	*p* value for interaction	0.90	0.26	0.25	0.25
	Body Mass Index				
	<24	−4.51 (−17.76, 8.75)	−4.86 (−11.68, 1.96)	3.45 (−6.7, 13.6)	−7.88 (−18.97, 3.21)
	≥24	−14.18 (−29.93, 1.58)	−2.11 (−8.35, 4.13)	−8.04 (−18.46, 2.39)	−4.72 (−15.06, 5.62)
	*p* value for interaction	0.84	0.64	0.40	0.78
	Cooking				
	Yes	−21.51 (−34.44, −8.58)	−7.24 (−12.59, −1.89)	−9.04 (−17.44, −0.63)	−13.15 (−22.03, −4.28)
	No	−11.19 (−35.66, 13.29)	−4.36 (−15.54, 6.82)	−3.53 (−22.16, 15.1)	−6.23 (−22.81, 10.35)
	*p* value for interaction	0.99	0.77	0.99	0.79
	Exercise				
	Yes	−18.09 (−32.96, −3.22)	−5.12 (−10.51, 0.27)	−6.94 (−15.6, 1.72)	−9.10 (−18.56, 0.36)
	No	0.25 (−17.31, 17.82)	−0.32 (−7.71, 7.08)	2.93 (−8.8, 14.66)	−2.15 (−14.24, 9.94)
	*p* value for interaction	0.20	0.18	0.55	0.22

Abbreviations: FVC, forced vital capacity; FEV_1_, forced expiratory volume in 1 second; NO_2_, nitrogen dioxide; O_3_, ozone; PM_10_, particulate matter <10 μm in diameter; PM_2.5_, particulate matter <2.5 μm in diameter. *Estimated change is calculated by linear regression models with adjustment for age, height, body mass index, passive smoking status, asthma, heart diseases, physical activities and cooking. Associations with lung function are scaled per 10 μg/m^3^ increase in all the pollutants concentrations for each moving average.
