# Supplementary material for: Short-term Effects of Outdoor Air Pollution on Lung Function among Female Non-smokers in China
Source: Sci Rep. 2016 Oct 13;6:34947. doi: 10.1038/srep34947 (PMC5062123; doi:10.1038/srep34947)
Supplement: Supplementary Information [file srep34947-s1.pdf]

Supplementary information

Short-term Effects of Outdoor Air Pollution on Lung Function among Female Non-smokers in China

Yun Zhou[1, 2#], Yuewei Liu[3#], Yuanchao Song[1,2], Jungang Xie[4], Xiuqing Cui[1,2], Bing Zhang[5], Tingming Shi[3], Jing Yuan[1, 2*], Weihong Chen[1, 2*].

Affiliations:

[1]Department of Occupational & Environmental Health, School of Public Health, Tongji Medical College, Huazhong University of Science and Technology, Wuhan, Hubei 430030, China

[2]Key Laboratory of Environment and Health, Ministry of Education & Ministry of Environmental Protection, and State Key Laboratory of Environmental Health (Incubating), School of Public Health, Tongji Medical College, Huazhong University of Science and Technology, Wuhan, Hubei 430030, China

[3]Hubei Provincial Key Laboratory for Applied Toxicology, Hubei Provincial Center for Disease Control and Prevention, Wuhan, Hubei 430079, China

[4]Department of Respiratory and Critical Care Medicine, Tongji Hospital, Tongji Medical College, Huazhong University of Science and Technology, Wuhan, Hubei 430030, China

[5]Department of Immunization Program, Zhejiang Provincial Center for Disease Control and Prevention, Hangzhou, Zhejiang 310051, China

# These authors contributed equally to this work.

Corresponding author:

Dr. Weihong Chen

Department of Occupational and Environmental Health

School of Public Health, Tongji Medical College

Huazhong University of Science and Technology

Wuhan, Hubei 430030, China

Tel: +86 27 83691677

E-mail: wchen@mails.tjmu.edu.cn

***Co-corresponding author:**

Dr. Jing Yuan

Department of Occupational and Environmental Health,

School of Public Health, Tongji, Medical College

Huazhong University of Science and Technology

Wuhan, Hubei 430030, China.

Tel: +86 27 83693209; E-mail: jyuan@tjh.tjmu.edu.cn

Supplement information

Supplementary Figure S1. Associations between moving averages of air pollutant exposures before test and lung function (N=1,694). Abbreviations: FVC, forced vital capacity; FEV_1, forced expiratory volume in 1 second. The linear mixed models included city (Wuhan and Zhuhai) as a random effect and adjusted for age, height, body mass index, passive smoking status, asthma, heart diseases, physical activities and cooking meals at home. Associations with lung function are scaled per IQR increase in all the pollutants concentrations for each moving average.

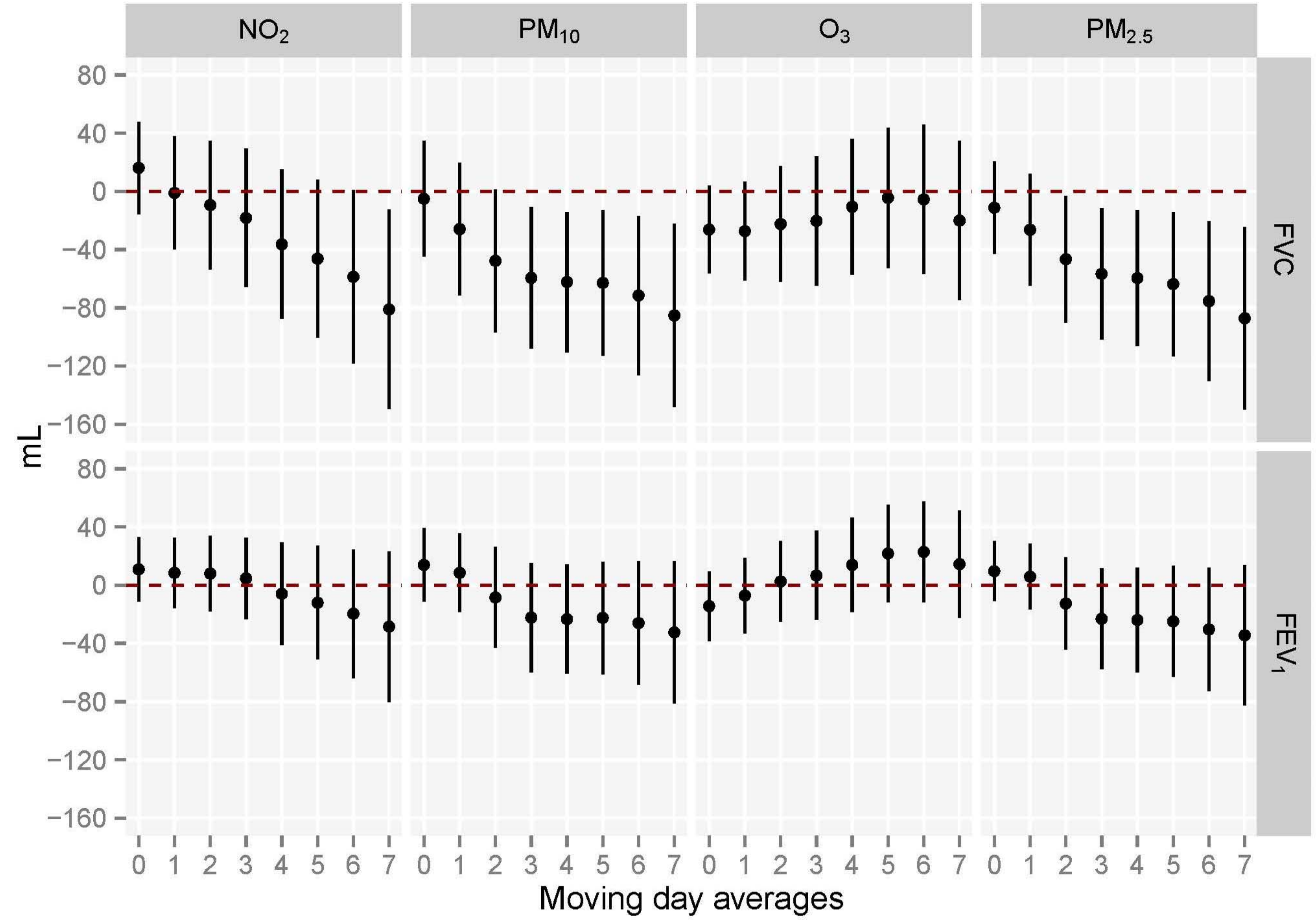
NO2
PM10
O3
PM2.5
FVC
FEV1
mL
80
40
0
−40
−80
−120
−160
0 1 2 3 4 5 6 7
Moving day averages

Supplementary Table S1. Distributions of the moving average of pollutant exposures among all participants (N=1,694)

Air pollutants	Distribution of all pollutants				Spearman correlation coefficients			
	Mean	SD	Range	Median	NO_2, μg/m^3	O_3, μg/m^3	PM_{10}, μg/m^3	$PM_{2.5}$, μg/m^3
Lag0								
NO_2, μg/m^3	54.66	34.15	7.75 to 118.00	48.2	1			
O_3, μg/m^3	103.20	43.08	32.00 to 193.00	107	0.49*	1		
PM_{10}, μg/m^3	128.36	65.39	48.00 to 274.00	128	0.71*	0.46*	1	
$PM_{2.5}$, μg/m^3	74.3	48.81	15.00 to 200.00	69.4	0.77*	0.49*	0.93*	1
Lag01								
NO_2, μg/m^3	55.72	31.36	8.50 to 108.00	53.8	1			
O_3, μg/m^3	101.63	38.16	43.28 to 182.55	95.6	0.64*	1		
PM_{10}, μg/m^3	127.78	60.79	45.50 to 242.00	129	0.76*	0.54*	1	

	$PM_{2.5}$, μg/m^3	73.33	44.18	16.75 to 179.50	64.6	0.75*	0.48*	0.96*	1
Lag02									
	NO_2, μg/m^3	55.84	29.22	9.40 to 103.33	56.27	1			
	O_3, μg/m^3	102.19	33.93	49.57 to 176.50	94.67	0.61*	1		
	PM_{10}, μg/m^3	126.10	57.14	43.92 to 222.67	129.33	0.79*	0.49*	1	
	$PM_{2.5}$, μg/m^3	72.04	40.78	17.37 to 154.53	62.2	0.79*	0.47*	0.96*	1
Lag03									
	NO_2, μg/m^3	54.76	26.97	10.05 to 100.00	54.6	1			
	O_3, μg/m^3	102.62	30.82	58.18 to 169.68	94.04	0.61*	1		
	PM_{10}, μg/m^3	122.18	54.49	41.69 to 215.00	111.5	0.79*	0.45*	1	
	$PM_{2.5}$, μg/m^3	69.62	38.41	17.98 to 143.05	59	0.82*	0.46*	0.97*	1
Lag04									

	NO_2, μg/m^3	54.78	24.91	11.59 to 95.28	55.28	1			
	O_3, μg/m^3	101.18	28.84	61.94 to 156.94	88.24	0.63*	1		
	PM_{10}, μg/m^3	118.57	52.64	44.71 to 214.00	105.2	0.82*	0.46*	1	
	$PM_{2.5}$, μg/m^3	67.56	36.4	18.33 to 141.08	61.88	0.86*	0.51*	0.95*	1
Lag05									
	NO_2, μg/m^3	54.71	23.27	14.09 to 98.73	53.67	1			
	O_3, μg/m^3	100.4	27.85	66.45 to 155.25	87.07	0.68*	1		
	PM_{10}, μg/m^3	114.88	49.85	46.53 to 209.67	100.33	0.83*	0.50*	1	
	$PM_{2.5}$, μg/m^3	65.53	33.87	20.08 to 138.48	59.13	0.89*	0.55*	0.95*	1
Lag06									
	NO_2, μg/m^3	54.92	21.15	20.54 to 97.20	55.94	1			
	O_3, μg/m^3	100.35	26.81	67.00 to 149.70	92.84	0.74*	1		

	PM_{10}, μg/m^3	112.45	45.76	47.57 to 204.29	98.71	0.83*	0.54*	1	
	$PM_{2.5}$, μg/m^3	63.90	30.90	24.49 to 134.21	56.43	0.90*	0.59*	0.95*	1
Lag07									
	NO_2, μg/m^3	55.74	19.22	24.98 to 93.95	58.65	1			
	O_3, μg/m^3	100.98	25.74	67.88 to 147.99	93.1	0.80*	1		
	PM_{10}, μg/m^3	111.34	41.84	48.73 to 202.25	104.13	0.85*	0.55*	1	
	$PM_{2.5}$, μg/m^3	63.28	28.19	26.20 to 130.01	59.06	0.90*	0.59*	0.96*	1

Abbreviations: NO_2, nitrogen dioxide; O_3, ozone; PM_{10}, particulate matter < 10 μm in diameter; $PM_{2.5}$, particulate matter < 2.5 μm in diameter; SD, standard deviation.

*p<0.01
